# 2D Materials for Cardiac Tissue Repair and Regeneration

**DOI:** 10.3389/fcvm.2022.802551

**Published:** 2022-02-11

**Authors:** Cemile Gokce, Cansu Gurcan, Lucia Gemma Delogu, Acelya Yilmazer

**Affiliations:** ^1^Department of Biomedical Engineering, Ankara University, Ankara, Turkey; ^2^Stem Cell Institute, Ankara University, Ankara, Turkey; ^3^Department of Biomedical Sciences, University of Padua, Padua, Italy

**Keywords:** two-dimensional nanomaterials, conductive biomaterials, cardiovascular disease, stem cell therapy, cardiovascular regeneration

## Abstract

Cardiovascular diseases (CVDs) have a massive impact on human health. Due to the limited regeneration capacity of adult heart tissue, CVDs are the leading cause of death and disability worldwide. Even though there are surgical and pharmacological treatments for CVDs, regenerative strategies are the most promising approaches and have the potential to benefit millions of people. As in any other tissue engineering approach, the repair and regeneration of damaged cardiac tissues generally involve scaffolds made up of biodegradable and biocompatible materials, cellular components such as stem cells, and growth factors. This review provides an overview of biomaterial-based tissue engineering approaches for CVDs with a specific focus on the potential of 2D materials. It is essential to consider both physicochemical and immunomodulatory properties for evaluating the applicability of 2D materials in cardiac tissue repair and regeneration. As new members of the 2D materials will be explored, they will quickly become part of cardiac tissue engineering technologies.

## Introduction

The field of biomaterials involves different scaled materials from macro-size to micro- and nano-sized materials categorized into polymers, ceramics, metals, and composites. Biomaterials work in conjunction with the living matter, directly replacing or restoring the damaged parts of the body that are tissues or organs by performing intended body functions and thus improving the quality of human life. They can exist naturally or be synthesized with specific functionality. Over the past few decades, they have been used in a variety of biomedical applications such as dental and orthopedic implants, sutures, joint replacements, heart valves, vascular grafts, contact lenses, pacemakers, burn dressing as well as drug delivery systems, and biosensors (1, 2). These applications rely on significant parameters such as biocompatibility, non-toxicity, host response (bioresorbable, bioinert, or bioactive), corrosion, wear, fatigue, design, and manufacturability. For example, polymers comprise the largest class of biomaterials and can be both natural and synthetic. They are covalently-bonded and mostly nonconducting materials used in drug delivery and soft and hard tissue applications. On the other hand, ceramics like calcium phosphates (CaP) and alumina (Al_2_O_3_) are characterized via ionic bonds, excellent biocompatibility, high corrosion resistance, wear, strength, hardness, and stiffness. However, metals such as stainless steel and titanium have great strength, resistance to fracture toughness, better elasticity, and rigidity compared to polymers and ceramics. They are mostly used in dental and orthopedics applications (3).

**Figure 1:**
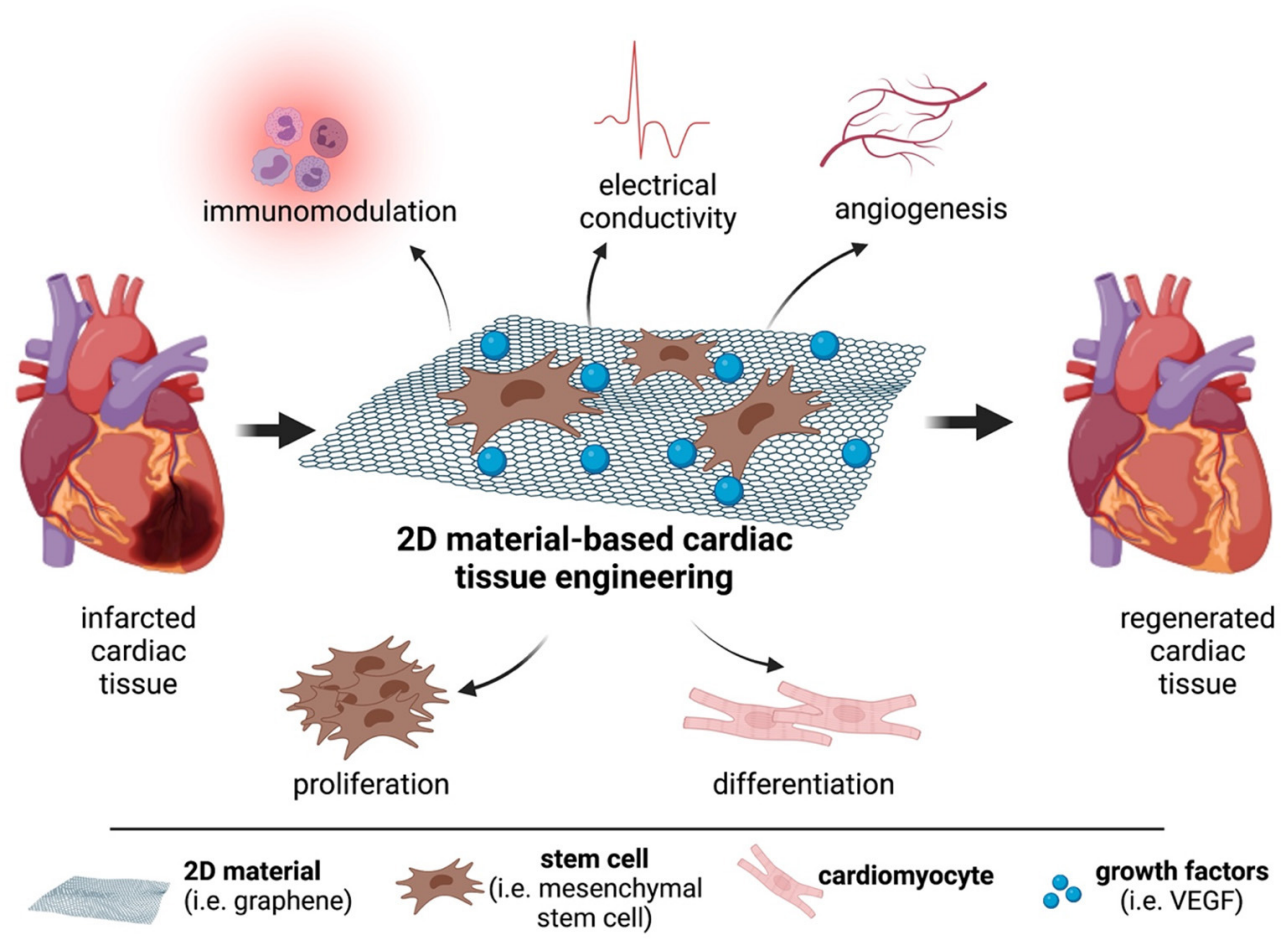
The potential of 2D materials for cardiac repair and regeneration. Graphene or graphene derivatives can act as scaffolds for mesenchymal stem cells and deliver growth factors to the infarcted area. This strategy can allow the regeneration of the damaged cardiac tissue.

2D materials (2DMs) refer to materials that are composed of thin layers with a thickness of a single or few atomic layers (4). The known 2DMs in literature are graphene (Gr), graphene derivatives (GDs) like graphene oxide (GO) and reduced GO (rGO), black phosphorus (BP), transition metal dichalcogenides (TMDs) such as molybdenum disulfide (MoS_2_), and tungsten disulfide (WS_2_), layered double hydroxides (LDHs), hexagonal boron nitride (h-BN), and transition metal carbides (MXenes). They possess unique intrinsic physicochemical properties such as high surface to volume ratio, excellent mechanical properties, electrical conductivity, and surface functionality which can be suitable for use in tissue engineering applications such as repair and regeneration of damaged cardiovascular tissues (CVTs) (5, 6). The damage in the CVTs can be caused by fat accumulation, platelet aggregation, and formation of blood clots resulting in CVDs such as ischemic stroke and myocardial infarction (MI) (7). Adult human heart has very low regenerative capacity, therefore, any cardiac damage in middle-aged or older people results in heart failure (8, 9). CVDs are major health problems in the United States of America, affecting 85.6 million American lives. According to WHO, CVDs account for 17.9 million deaths in 2019, 32% of all global deaths (10, 11). For this reason, novel approaches are required to reverse the CVD progression. Thanks to their intrinsic properties, 2DMs are promising candidates which can overcome the limitations or improve the performance of conventional biomaterials or other available therapies (Figure 1). Scaffolds containing 2D materials can allow effective proliferation and differentiation of stem cells, and contribute toward the regeneration of damaged cardiac tissue through its angiogenic, immunomodulatory, and electrical properties. This review article introduces the current clinical therapeutic approaches for CVDs, and their limitations are discussed. Later, biomaterial and nanomaterial-based studies are explored with a specific focus on 2DMs to delineate their potential in the regeneration and repair of CVTs.

There are several adult stem cells such as hematopoietic stem cells (HSCs), mesenchymal stem cells (MSCs), cardiac stem cells (CSCs), and endothelial progenitor cells (EPCs) which are used in clinical applications of cardiovascular therapies (12). Among them, the treatment of CVDs with mesenchymal stem cells (MSCs) is a good candidate and is used commonly for tissue regeneration due to the immunomodulatory and vascular repair capabilities of MSCs. They are multipotent progenitor cells with self-renewing properties and are found in different tissues like adipose tissue, bone marrow, and umbilical cord blood as cell sources (13, 14). According to their sources, MSCs can exhibit different properties, including immunomodulatory and paracrine effects, and differentiation into specific cell types (15). For CVD, they have already reached pivotal phase III trials after successful completion of phase I and II clinical trials (7, 16). Prior to clinical trials, large animal models like pigs, dogs, and sheep are better at testing the feasibility, efficacy, and long-term safety of therapeutic approaches for cardiac regeneration due to their similarity to human physiology and anatomy. These models select an optimal number of transplanted cells and the time of transplantation to deliver and track transplanted MSC. Nevertheless, these models have a high maintenance cost and long-life cycle, unlike small animal models like rodents (17, 18). Even though MSCs are found not to initiate immunological reactions within 12 months post-transplantation in many clinical trials, they promote anti-inflammatory actions (19–21). Bone marrow-MSCs are commonly used in clinical trials of acute/subacute MI and found to be safe for a small group of patients who had acute MI at short and long-term follow-up (22). Trials with adipose tissue-MSCs have been performed to test their efficacy in myocardial regeneration. It has been shown that adipose tissue-MSCs enhance cardiac function and decrease scar tissue (23). Additionally, umbilical cord Wharton's jelly (WJ)-derived MSCs express higher proliferative potential, safe, beneficial, and feasible in trials for treating acute MI (15, 24, 25). In another trial with autologous MSCs, therapeutic effects such as decrease in scar tissue, increase in heart mass, and contractility have been successfully observed (26, 27). Later, allogeneic and autologous bone marrow-MSCs are compared for the treatment of chronic ischemic cardiomyopathy (CIC). Both allogenic and autologous cells show similar results, including a decrease in scar size and enhancement of ejection fraction (28). Bone marrow-MSCs cause a decrease in infarct size and improvements in contractility, although no change in ejection fraction was observed (29). Likewise, administration of autologous MSCs into patients undergoing cardiac surgery results in decreased scar size, improvements in contraction, and tissue perfusion (30). Stem cell-based therapies based on bone marrow-derived MSCs can also improve heart regeneration via transplanting non-cardiac cells because there are not enough functional cardiomyocytes to replace the lost myocardium (28, 31, 32). Adipose tissue-MSCs are also tested for treating seriously ill CIC patients and show improvements in heart contraction, perfusion, and left ventricle mass (33). In a slightly different approach, MSCs are stimulated with cardiogenic cytokines before transplantation in order to improve therapeutic efficacy (34). A similar strategy is performed by stimulating adipose tissue-MSCs with vascular endothelial growth factor (VEGF) (35). In conclusion, MSCs generally improve the quality of life by decreasing the size of scar tissue, increasing contractility and tissue perfusion of the injured heart, inducing the formation of new blood vessels, and antifibrotic effects at the damaged cardiac tissue. However, these therapies have major limitations, including poor *in vivo* survival rate after transplantation, and insufficient adult stem cell delivered to the damaged target area (7, 36, 37). The small number of participants and the lack of a placebo group in clinical trials may also restrict the therapy's positive generalized effect, such as long-term safety. These limitations may be overcome by priming MSCs with stem cell modulators used for intracellular signal triggering prior to transplantation or via alternative strategies such as scaffolds involving natural polymers or nanomaterials (9, 38).

## Biomaterials in Cardiovascular Regeneration

Current cardiac therapies face challenges such as the minimal intrinsic regenerative capacity of heart tissue, poor integration, and implanted cell survival (39). Thus, there is an urgent need for cheaper and less invasive cardiac treatment approaches via biomaterials or nanomaterials. The biomaterials-based approach is one of the most plausible ways to attain clinically successful cardiac therapies. Biomaterials used in cardiac repair should be biocompatible, biodegradable, enable cell integration with native tissue, decrease hostility of the local microenvironment and control the slow release of bioactive molecules (40–42). Both synthetic biomaterials like polylactic-co-glycolic acid (PLGA), polyurethane, and carbon nanotubes (CNTs) and natural biomaterials like fibrin, gelatin, alginate, collagen, and chitosan can be used alone as a carrier or serve as an artificial extracellular matrix (ECM) scaffold to repair and regenerate CVTs (42–44). The myocytes and other cells in the heart interact with the ECM, which comprises inotropic stimuli and growth signals for cell recruitment and maturation (45). Other cells in the heart are stem cells like mononuclear cells (MNCs), embryonic stem cells (ESCs), bone marrow-MSCs, and/or progenitors like EPCs, and transplantation of these cells mainly provides the formation of new and functional myocardial tissue. These cells can be mixed with biomaterials for direct delivery to the target area or cultured on biomaterials (46, 47). This mixing or culturing indicates better transplantation, survival, differentiation, and functioning of the cells in the heart and thus further development in cardiac tissue regeneration. Besides, the materials for cell delivery should have an optimal degradation time for the electromechanical integration of transplanted cells (48, 49). Growth factors such as transforming growth factor-β (TGF-β) and bone morphogenetic protein 4 (BMP4) play an essential role in mediating cardiac tissue regeneration. Based on this knowledge, the therapeutic potential of injectable biomaterials like hydrogels against MI and peripheral artery disease (PAD) has been investigated in animal models (50). *In vivo* injection of keratin biomaterials and platelet gels can provide sustained release of growth factors which preserved cardiac tissue, promoted angiogenesis, and decreased left ventricular wall dilatation. Their porous structure also allows the endothelial cells, cardiomyocytes, and other progenitor cells to grow on the scaffold, making them good candidates for cardiac repair (44, 51). Solid forms of alginate, a naturally occurring polysaccharide found in algae, like hydrogels and microporous scaffolds, are used for biomaterial-assisted stem cell transplantation for the failing cardiac tissue (52). This transplantation replaces lost stem cells in the heart and encourages native cardiac tissue regeneration. Concerning this, Levit et al. have designed a hydrogel delivery platform for stem cell transplantation. They have encapsulated human MSCs (hMSCs) in an alginate hydrogel. After encapsulation, MSCs have been attached to the damaged rat heart with a PEG hydrogel patch for stem cell protection. The study results have concluded that alginate encapsulation enhances retention of hMSCs and facilitates paracrine effects such as increased microvascular density and reduced scar size, providing cell-mediated cardiac repair (53). Roche et al. have carried out a study to evaluate whether acute retention can be improved with biomaterial carriers. They have used a thermosensitive chitosan/β-glycerophosphate gel, alginate, and collagen patches as the hMSC vehicles, chitosan hydrogel as a delivery vehicle for adipose tissue-MSCs in myocardial repair. They have displayed promising results that all studied biomaterials have influenced the part where cells are localized and improved retention of cells in the myocardium (54). Biomaterials, especially polymers including fibrin, alginate, collagen, poly(L-lactic acid) (PLLA), and poly(glycerol sebacate) (PGS) with inherent characteristics have also been used to control the differentiation process of induced pluripotent stem cells (iPSCs) as well as increasing the quality and efficiency of their traditional expansion to get an advanced iPSC research. They do this by creating the native microenvironment of stem cells, namely stem cell niche (55, 56).

In addition to biomaterials, nanomaterials in the forms of nanostructured surfaces, nanoparticles, and nanocomposites have unprecedented potential to enhance the therapeutic performance of conventional biomaterials. These nanomaterials can be gold nanoparticles (Au NPs), silver (Ag) NPs, polymeric NPs, cerium oxide (CeO_2_) NPs (nanoceria). Their size ranges from protein level to cellular level and can mimic the native ECM components of tissues and the cardiac microenvironment (57–59). They have physiochemical properties such as high surface-to-volume ratio, surface roughness, hydrophilicity, high surface energy, and reactivity, promoting better protein adhesion and direct cell activities. This ability to direct cell fate selectively is useful in specific cardiovascular studies where it is preferable to stimulate the functions of some cell types while suppressing the activities of another cell type (60–62). They cause oxidative stress by producing ROS, which is one of the main players of inflammation. Moreover, integrating nanoscale components of different sizes, morphology, function, and chemical composition into a matrix material is also a more reliable approach to obtain complementary properties required for effective therapy. Briefly, they can either induce immune suppression or stimulation depending on their size, surface chemistry, composition, protein binding, and exposure route (59, 63). Immunosuppression occurs when the functioning of the immune system disrupts and thus causes non-effective clearance of pathogens, whereas immunostimulation enhances the response of the immune system specifically to resist pathogens (64). For instance, Nanoceria has been found to uptake by the cardiac progenitor cells (CPCs), reduce ROS as an antioxidant agent, and protect the cardiac progenitor cells from H_2_O_2_-induced cytotoxicity (65). It has also suppressed the activity of the innate immune system in *Paracentrotus lividus (P. lividus)*, Mediterranean species of the sea urchin (66). Mukherjee et al. have developed a PLLA/poly(ε-caprolactone)/collagen nanostructured matrix mimicking the native myocardium microenvironment. This hydrophilic nanofibrous biocomposite scaffold has been used to culture and support isolated rabbit cardiomyocytes. They have demonstrated superior attachment and growth of adult rabbit cardiomyocytes (67). Chang et al. have conjugated insulin-like growth factor (IGF)-1 to PLGA nanoparticles and delivered to the damaged area after acute MI. In this study, the complex has increased IGF-1 retention, improved left ventricle function, and provided early cardioprotection (68). Martins et al. have described electrically conductive CNTs/chitosan scaffolds as having similar mechanical properties to native cardiac muscle. The scaffold has supported the survival of neonatal rat cardiomyocytes *in vitro* for up to 14 days and increased the expression of cardiac genes important for muscle contraction, electrical coupling, and cell-to-cell signal transmission (69). Similarly, Zhou et al. have developed a scaffold made up of single-walled CNTs and gelatin hydrogel to support culturing of neonatal rat cardiac cells *in vitro*. This scaffold was able to inhibit pathological deterioration of the myocardium and enhance cardiac function post-MI (70). Other clinical studies highlighted the importance of having alginate-hydrogel for treating advanced heart failure compared to standard medical therapy. Alginate hydrogel was shown to be more effective than standard medical therapy for improving exercise capacity, symptoms, and clinical status (71, 72). In a study by Meslmani et al., PLGA nanoparticles were immobilized on polytetrafluoroethylene (ePTFE) films to develop vascular grafts as a potential vehicle for antithrombotic drugs reducing side effects of thrombosis (73). Ahadian et al. have incorporated polyester with CNTs to make a polymeric scaffold for cardiovascular studies, and they have found that this approach increases the stability and electrical conductivity of the polymeric scaffolds (74). Liu et al. have introduced titanium dioxide (TiO_2_) nanoparticles into the polyethylene glycol (PEG)ylated chitosan hydrogel matrix for CVT repair. This nano-modified hydrogel has induced cell adhesion, organization of cardiomyocytes and improved mechanical and swelling behaviors (75). Finally, Au NPs have been also explored for cardiac tissue engineering due to their numerous advantages, including biocompatibility, chemical stability, intrinsic optical properties, ease of surface functionalization, anti-cardiac hypertrophy effect, high electrical conductivity, and availability in various ways of geometries (i.e., nanospheres, nanorods, nanowires). For example, combining Au NPs with ECM/silk fibroin has resulted in favorable conductivity, retention of cardiomyocyte survival, increased cell compatibility *in vitro*, and decreased infarct size *in vivo* (76). To conclude, several types of biomaterials and nanomaterials offer great opportunities to face challenges related to the treatment of CVDs.

## The Potential of 2D Materials in Cardiovascular Regeneration

CVDs which include the main pathologies such as ischemic heart disease (IHD), stroke, and peripheral ischemic disease, are priority areas for tissue regeneration (77). The current conventional device-based or pharmacological therapies cannot reverse the myocardial function loss. They are limited by the different disease presentations of patients with conduction disorders, such as atrioventricular (AV) block and ventricular arrhythmias (78). As an example, an electronic pacemaker controlling heart pumping function reduces patient mortality and hospitalization, but, the conduction blockage is not solved because non-conductive tissue or an abnormal conductive pathway still exists in the cardiac tissue (42, 79). For this reason, conductive biomaterials or nanomaterials could be integrated with the pacemaker to exhibit high electrical conduction of these abnormal ventricle functions (80–82).

Recently, researchers have been investigating comparatively new 2DMs-based applications for cardiac tissue engineering. To illustrate, Gr is a good candidate for regenerating different tissues with its high specific surface area, porosity, antibacterial activity, and excellent mechanical strength. Gr has been proven to guide and accelerate the differentiation of stem cells like MSCs into specific cell types, including adipocytes, osteoblasts, and chondrocytes, through interactions with chemical inducers such as insulin, which mediates fatty acid synthesis and adipogenesis. Just as in Gr, GO directs stem cell differentiation. This is mainly occurred due to the functional groups on the surface of GO that adsorb ECM proteins. This adsorption of ECM proteins on GO promotes cell adhesion on its surface (83, 84). In 2016, many studies about the immunomodulatory effect of Gr were also reported (85). As an implantable biomaterial and scaffold, it interacts with host tissue cells, exerts an intense impact on the immune response of that host, and thus affects cardiac repair. In general, the host innate immune system consists of phagocytic cells such as macrophages, neutrophils, and dendritic cells (DCs). They are introduced as the first line of defense against foreign particles or microorganisms and maintain tissue homeostasis. Gr encounters the cells of the immune system, firstly macrophages secrete inflammatory cytokines like interleukin (IL)-6, and IL-8, and pro-angiogenic factors such as VEGF, and tumor necrosis factor-alpha (TNF-α). In CVDs, macrophages enhancing pro-inflammatory cytokines are classically activated and thus can be classified under the M1 phenotype. However, they are polarized into alternatively activated M2 phenotypes during cardiac treatment (86). Thus, the general issue is to have novel biomaterials as agents utilizing and modulating the power of macrophages to get an appropriate immune response (87, 88). Malanagahalli et al. prepared a few-layer Gr (FLG) and investigated its biological response on mouse bone marrow-derived macrophages (BMDMs). They found that increased doses of FLG did not hinder the viability of cells, caused no significant secretion of pro-inflammatory cytokines such as IL-6 and TNF-α, and did not evoke inflammatory responses in primary BMDMs (89). It is of interest to note that the current studies to regulate cardiac inflammation include the targeting of other immune cells which take part in adaptive immunity like B cells, and T cells or factors including reactive oxygen species (ROS) (90, 91). To exemplify, Tomić et al. have investigated the immunomodulatory actions of Gr quantum dots (GQD) in human peripheral blood MNCs. They showed that GQD inhibits the proliferation of the MNCs, reduces the functions of monocyte-derived DCs and the proliferation of T cells while augmenting the production of anti-inflammatory cytokines that are advantageous in studying T cell-mediated pathologies (92). Moreover, the current *in vitro* cell differentiation methods, particularly iPSCs derivation, expansion, and differentiation, do not completely mimic the structural properties such as myofibril organization and electrophysiological properties such as conduction velocity of cardiomyocytes. To overcome this, Wang et al. have maintained human iPSCs (hiPSCs) lines on Gr sheets and demonstrated that Gr sheets with a conductive surface can improve the maturation of hiPSCs derived cardiomyocytes without the need for electrical stimulation. This facilitates cell to cell communication via improved expression levels of connexin 43, a gap junction protein (93). Other GDs especially GO hold great mechanical properties similar to natural cardiac tissues that are undoubtedly suitable for CVT engineering and have been extensively studied in the literature. Accordingly, Park et al. have studied the effects of GO attachment on MSCs prior to MSC transplantation into the infarcted myocardium. This attachment of GO to MSCs protects interactions between MSCs and ECM proteins against ROS-mediated deterioration of cell adhesion and then maintains the survival of MSCs. The transplantation of stem cells without the aid of GO will not be able to result in interaction with ECM owing to the ROS generated in the ischemic myocardium and will eventually die. Thus, this study is highly essential to understand the importance of MSC transplantation with 2D materials (94). Wang et al. have prepared complexes of GO with polyethyleneimine (PEI) or PEG to examine their interactions with mouse monocyte-macrophage cells. As a result, both GO-PEI and GO-PEG have very low cytotoxicity toward the mouse cells, and GO-PEG gives rise to a stronger immune response, while GO-PEI displays no obvious stimulation (95). Paul et al. made a nanocomposite hydrogel composed of functionalized GO, DNA_VEGF_, and gelatin methacryloyl (GelMA) in order to evaluate its therapeutic efficacy for MI *in vivo*. Authors have shown that the injected hydrogel has enhanced contractile performance, and the injured tissue was repaired without cytotoxicity (96). Bao et al. have combined conductive hydrogel with GO and adipose tissue stem cells (ADSCs) to form PEG/melamine (MEL)/thiol modified hyaluronic acid (HA-SH) hydrogel. Following its injection into the MI region in a rat model, authors have observed smaller infarct size, thicker left ventricular wall, and higher expression of cardiac genes, α-smooth muscle actin (α-SMA) and Cx43, after treatment (97). Saravanan et al. have highlighted that the implantation of GO-Au nanosheets together with chitosan scaffold improves ventricular contractility and function (98). Choe et al. have devised a reduced GO/ alginate microgel to obtain higher therapeutic efficacy and functional infarcted cardiac tissues. They have suggested that the microgel could scavenge ROS, which allowed cardiomyocytes to continue living and functioning even when exposed to H_2_O_2_ (99). Nazari et al. have developed polyurethane (PU)/rGO-Ag nanofibrous scaffolds, which were able to upregulate specific cardiac genes like alpha-MHC, GATA-4, T-box 18, and cTnT (100). Other 2D materials have also been reported to be used in cardiovascular repair and regeneration. For instance, Nazari et al. have combined MoS_2_ nanosheets with nylon6 nanofibers for cardiac repair. Such an approach has resulted in higher conductivity and better biocompatibility than pure nylon6 (101). In another study, Ti_3_C_2_T_x_ MXene nanosheets have been shown to create physiologically relevant cardiac patches (CPs) for the treatment of MI (102). In another similar study, MXene Ti_2_C was incorporated into a cryogel resulting in a conductive CP, which was shown to promote functional maturation of cardiomyocytes and enhance repair of myocardial infarction (103). Following the applications of MXenes in the field, MXene quantum dots (QDs) also showed potential in CVT regeneration. In 2019, injection of a hydrogel containing Ti_3_C_2_T_x_ MXene QDs at an infarct site was shown to reduce recruitment of selected immune cells, which are human T-lymphocytes, as well as promote immunosuppressive regulatory T cells, and limit secondary injury to the heart (104). More recently, the same group immune-engineered tantalum carbide (Ta_4_C_3_T_x_) MXene QDs to develop immunomodulatory material that can inhibit desired cellular immune responses during the healing process via regulation of surface co-activator and co-inhibitor molecules. In their report, they have shown that these materials were able to ameliorate the cellular and structural changes of early allograft vasculopathy (105). Such an immune-engineering approach can overcome one of the biggest limitations of CVT engineering in the future.

Overall, Gr and GDs are among the most popular 2D nanostructures in cardiovascular repair and regeneration according to the literature. Although other 2DMs are being used in bone, skin, and neural tissue regeneration and wound healing, they are attractive to researchers in cardiac tissue therapy (6). Based on their physicochemical characteristics, 2DMs possess intrinsic immunological activities useful in cancer immunotherapy, chronic inflammation, organ transplantation, and autoimmune diseases. They can be recognized by the immune cells, resulting in the activation or suppression of the immune system (85). They can also serve as antioxidant agents preventing ROS-mediated cell stress and death (106). As reported by Gazzi et al., various groups have spent efforts to obtain qualitative and quantitative immune characterization and to define immune profiles of Gr, GDs, and other carbon-based nanomaterials (107). Furthermore, the interactions of GDs or MXenes with immune cells have been demonstrated as advantageous for tissue regeneration. Thus, it is essential to consider the physicochemical characterization and immunomodulatory properties for evaluating the applicability of 2D materials in cardiac tissue engineering.

## Future Perspectives

CVDs emerge and affect many people worldwide, and thus cardiovascular repair and regeneration via cell therapy have progressed within the past decade. Many researchers have studied and tested the efficacy of using stem cells together with biocompatible biomaterials or nanomaterials to treat CVDs (108–110). Over the last years, unique 2DMs showing similar electrical and mechanical properties to native myocardium have been studied to achieve successful therapies. However, *in vivo* behavior and interaction mechanism of 2DMs with biomolecules, degradation profile, long-term safety, and hard-to-control fabrication process have not been fully optimized yet (4, 6). On the other hand, various groups have emphasized the importance of material properties which directly determine their behavior in the biological environment, including toxicity and degradation profiles (111). For this reason, by controlling surface chemistry, shape, and size, we can make them safe and effective helpers of cellular therapies for CVDs. All materials mentioned in this review harbor important properties for cardiac tissue repair and regeneration. Table 1 summarizes different studies performed in the field, and discusses advantages and limitations of each study. Each of these materials could improve a certain function, even if they sometimes elicit multiple actions. In particular, among 2D materials, Gr and GO have been shown to be potential platforms for stem cell delivery, adhesion, growth, and differentiation through their adsorption capacity for hydrophobic and electrostatic interactions. BP has been reported to be highly electrically conductive, induce a low-ROS microenvironment, and restore angiogenesis, as well as neurogenesis, but no studies have reported its integration and the direct action mechanisms on cardiomyocytes. Whereas, another 2D material MoS_2_ is used for its good cytocompatibility and its ability to conduct electrical signals. MXenes are used for the modulation of the immune response, and improvement of cardiac differentiation.

**Table 1:** 2D material-based studies in cardiovascular tissue engineering.

	**Composition**	**Model**	**Cell**	**Disorder**	**Advantage**	**Limitation**	**References**
Gr	With and without oxygen plasma	*in vitro*	hiPSCs-derived cardiomyocytes (CMs)	Not specified	* Low cost, robust, and flexible * Biomimetic conductive surface * Increased Cx43 expression and electrical propagation * Enhanced Ca^2+^ handling * Effective hiPSCs differentiation into CMs, and CMs maturation * Increased BMP signaling during cardiogenesis *Cx43 = cardiac gene important for electrical conduction	* Reduced conductivity and elevated BMP signaling via oxygen plasma modification * Only two-fold increase in conduction velocity, correlated with Cx43 gene expression	(93)
GO	None	*in vitro* ROS presentation and *in vivo* implantation	MSCs	Myocardial ischemia	* Non-significant toxicity * Enhanced MSCs adhesion via ECM protein adsorption on GO * Increased MSCs survival rate even under ROS presence * High number of engrafted MSCs * Improved paracrine factors secretion from MSCs * Reduced cardiac tissue apoptosis by promoting angiogenesis * Improved cardiac therapy	* Long-time retention of GO post-implantation *in vivo*	(94)
	poly-L-lysine (PLL)	*in vitro*	3T3 fibroblasts, CMs, ECs, and hMSCs	Not specified	* Suitable interface material * Strong cell-cell electrical coupling between layers via the adhesive thin film structure * Strong spontaneous beating * Stably organized 3D-engineered cardiac tissue * Improved cardiac cell maturation * Enhanced biological activity via PLL and mechanical integrity via GO	* Decreased conductivity with PLL functionalization at the beginning (before its degradation) * Decreased cell viability with increased number of layers * Cytotoxicity of cationic PLL at high concentrations	(112)
	PEI/DNA_VEGF_/GelMA	Both *in vitro* and * *in vivo* (rats)	HUVECs, and embryonic rat CMs (H9c2)	Acute MI	* Biocompatible * Non-significant toxicity even in long-time post-administration * Supported microvasculature and endothelial cord formation via GelMA * Effective *in vivo* gene transferring via PEI * Proangiogenic activities via DNA_VEGF_ in vitro * Increased myocardial capillary density * Reduced scar size	* Unknown salutary effects of multiple administration of the functionalized GO on cardiac functioning	(96)
	PEG-MEL/HA-SH	Both *in vitro* and *in vivo* (SD male rats)	ADSCs	MI	* Superior therapeutic efficacy with very high cytocompatibility * Very soft mechanical property via flexible PEG * Anti-fatigue, stable, and conductive property close to native myocardium via GO * Faster gelation process via PEG-MEL/HA-SH * Resistant to dynamic stress * Effective electromechanical signal transmission with cell-cell communication, * Enhanced Cx43 and α-SMA expression * Higher vessel density and ejection fraction * Reduced fibrosis area * Thicker left ventricular wall	* A comparatively small reduction in the infarction size than the study by Wang et al. (113)	(97)
	OligoPEG fumarate (OPF)	Both *in vitro* and *in vivo* (rats)	Mouse ESCs, CMs	MI	* Improved *in vitro* cell attachment * Enhanced mechanical support, cytoskeletal structure and electrical signal propagation * Enhanced Ca^2+^ signal conduction of CMs in the infarcted area * Better heart functioning via increased intercalated disc formation * Promoted Cx43 expression, and macrophage activation with increased CD68+ cells ratio	* GO alone may not be used as a carrier of mouse ESCs * OPF alone is non-conductive, which may not restore impulse propagation	(114)
	Au-Chitosan	*in vitro*, ex-vivo and *in vivo* (male Wistar rats)	iPSCs-derived CMs, rat smooth muscle cells, mouse fibroblasts,	MI	* Two-fold increased conductivity via Au * Enhanced conduction velocity and cardiac contractility in infarcted area * Restored ventricular function * No immune response in the myocardium (unchanged numbers of * CD4+ and CD8+ T cells)	* Lack of electrical conductivity in chitosan * Aggregation of Au in the chitosan microstructures, causing stress and damage on tissue	(98)
	Chitosan	(1) *in vitro*	hESC-derived fibroblasts, and CMs	Not specified	* Enhanced cell viability and proliferation * Rapid self-healing property	* Very brittle pure chitosan, * Decreased adhesive strength of GO at 0.75 and 1 mg/mL	(115)
		2)*in vitro*	H9c2		* Swelling, porosity, and conductive properties * Increased cTnT expression as well as Cx43 without exogeneous electrical stimulation, Large amounts of ECM secretion * Supported cell adhesion and extension *cTnT= cardiac Troponin T, gene important for contractile function	* Decreased porosity and cell viability with GO concentration in 600 mg/L of the scaffold	(116)
	Polyethylene terephtha-late (PET)	*in vitro*	HUVECs, and H9c2	Not specified	* Potential electroconductive CP * Almost two-fold increase in mechnical behavior * Improved wettability * Controlled cellular behaviors, cell attachment, and spreading * Guaranteed cardiac cell support for ~2 months	* Unsupported elongated cell morphology of PET without GO * Unmonitored long-term degradation rate and process of the CP	(117)
	Reverse Thermal Gel (RTG)	*in vitro*	Neonatal rat ventricular myocytes (NRMs)	Not specified	* Temperature-dependent changes from 2D gelation to 3D matrix gel * Negligibly invasive system with low viscosity * Better cell proliferation, alignment, maturation and long-time survival * Non-cytotoxicity	* High conducting resistance of RTG alone * Non-increased number of α-actinin positive cells until 7^th^ day of culturing	(118)
	Gelatin	*in vitro*	NRMs	Not specified	* Long-term functional CM culturing * Stiffness similat to native myocardium via GO * Longest sarcomere length on 3rd day culturing * Promoted beating velocity via GO * Better cell alignment and synchronous contraction via micropatterning	* Very soft, physically non-stable, and thermosensitivity properties of gelatin alone	(119)
rGO	Fibronectin	Both *in vitro* and *in vivo* (mouse)	MSCs	MI	* Enhanced cell-ECM interactions, angiogenic growth factor expression, and Cx43 upregulation * Increased MSC function via electrical conductivity of rGO * Higher ejection fraction and better cardiac performance *in vivo*	* Limited cell-ECM interactions, and growth factor expression in MSCs alone * Induced cell stress and apoptosis with GO above 10 μg/mL	(120)
	GelMA	*in vitro*	rat CMs	Not specified	* Higher cell retention, stronger contractility and faster spontaneous beating rate compared to GO-GelMA	* Limited thickness of the functionalized hydrogel via rGO concentration	(121)
	PU and Ag	*in vitro*	hCPCs	Not specified	* Upregulation of GATA-4, T-box 18, and cTnT * Increased tensile strength, wettability, and electrical conductivity via rGO-Ag * Induced cardiogenic differentiation via rGO	* Non-efficient electrical conductivity of PU alone * Cytotoxicity of Ag without rGO	(100)
	Alginate	Both *in vitro* and *in vivo* (rats)	MSCs, and neonatal rat CMs	Acute MI	* High cell protection capacity * Increased cell viability under H_2_O_2_ stress via superior antioxidizing activity of rGO * Enhanced MSCs delivery, and cardiac function	* Low viability and function of MSCs alone due to harsh conditions	(99)
	Collagen	*in vitro*	HUVECs, and CMs	Infective endocarditis (IE)	* Electroactive CP * Electrical coupling (Cx43), muscle contraction, relaxation (cTnT) and cytoskeleton alignment (α-actinin4) after 7 days * Promising antibacterial activity for preventing IE	* Decreased pore size of collagen scaffold and possible cytotoxicity with increased rGO concentration	(122)
	Polyester amide (PEA)-Chitosan	*in vitro*	10 T1/2 cells, and iPSC-derived MSCs	Not specified	* Increased PEA porosity via ultrasonication and leaching of PEO/PEA * Lowering of the required voltage for electrospinning via rGO * Supported cardiac differentiation * Non-cytotoxicity	* Limited cell infiltration and tissue maturation of electrospun PEA alone * Slightly less conductive effect of PEA-rGO with chitosan	(123)
	Gellan gum	*in vitro*	H9c2	Not specified	* Higher compressive modulus, and water-swelling ratio via rGO * Suitable injectability * Promising candidate of cardiac repair with 2wt% rGO	* Insignificant increase in compressive strength, ductility, and degradation	(124)
TMDs	MoS_2_/Nylon6	*in vitro*	Mouse embryonic cardiac cells (mECCs)	Not specified	* Better cell elongation and differentiation on the scaffold via MoS_2_ * Upregulation of cardiac genes, which are GATA-4, c-TnT, Nkx 2.5 and α-MHC *~4 fold increase in electrical conductivity via MoS_2_ * Mechanical support via Nylon6 * Increased fracture modulation and decreased modulus via MoS_2_	* Lack of electrical conductivity property of Nylon6 alone * Decreased Nylon6 wettability with MoS_2_ presence	(101)
MXenes	Ti_2_CT_x_/ PEGdiacrylate (PEGDA)/ GelMA (Ti_2_C-cryogel)	Both *in vitro* and *in vivo* (SD rats)	Rat aortic endothelial cells (rAECs), and neonatal rat CMs	MI	* Hydrophilic, elastic and conductive CP * Induced tube structure formation of ECs * Obvious left ventricle anterior wall activity * Recruited macrophages and released TGF-β * Improved cardiac function and scar formation * Promoted *in vivo* vascularization	* Very low conductivity of MA/Ti_2_C * Low mechanical strength and toughness of pure cryogel	(103)
	Ti_3_C_2_T_x_/PEG	*in vitro*	hiPSCs-derived CMs	MI	* 3D printed CP with high electroconductivity and fibrillar structure * Promoted cell viability, and alignment * Better coordinated cell network * Significant increase in MYH7, SERCA2, and TNNT2 expression with increased cell maturity * Improved beating cell area percentage, and conduction velocity * Low protein adsorption property of PEG	* Slightly decreased sarcomere length on the construct via Ti_3_C_2_T_x_ * Non-observable gene upregulation effect at the protein level * Insignificant improvement in the beating rate of the CMs	(102)
MXene QDs	Ti_3_C_2_T_x_/ Chitosan	*in vitro*	Bone marrow MSCs, and iPSCs-derived fibroblasts	Not specified	* 3D platform with stretchable, and flexible shape memory properties * Increased electrical signal transmission * Supported cell survival and proliferation * Maintained injectability and thermosensitivity * Promoted immune tolerance, and decreased T-cell dependent inflammatory response	* Smaller swelling degrees of Chitosan alone	(104)
	None (Ta_4_C_3_T_x_)	Both *in vitro* and *in vivo* (male Lewis and SD rats)	Antigen-presenting ECs (HUVECs)	Cardiac Allograft Vasculopathy (CAV)	* Biocompatibility and high electrical conductivity * Intrinsic anti-inflammatory and anti-apoptotic properties * Decreased expression levels of CD86, a co-activator to T-cell activation * Ameliorated loss of medial α-SMA * Reduced early development of allograft vasculopathy	* Uncertain infarcted cardiac tissue repair, only vascularization of descending thoracic aorta	(105)

For good therapeutic results, it is vital to find the best way of transferring cells along with the nanomaterials to the infarcted cardiac tissues without any or minimal conduction abnormalities. This can be achieved by considering and optimizing conditions such as duration of disease (acute or chronic disorder), the number of transplanted cells, sex, and patient age (9, 42). The matrix should resemble the myocardial architecture to control the maturation stages better and develop highly efficient models limiting scar tissue formation. For this purpose, studies have been performed with nanofibrous PLLA scaffolds for vascular regeneration. Human and mouse-derived iPSC-derived smooth muscle cells (SMCs) have been seeded onto a 3D macro-porous and nanofibrous PLLA scaffolds. This seeding process maintains the maturation of *in vitro* iPSC-derived SMCs by inducing functional contractile phenotype and promotes *in vivo* vascular structure formation (125–127). Furthermore, immense studies are required to face challenges related to selected cell types or combinations and electromechanical stimulation (41, 42). Apart from this, new engineering technologies might allow three-dimensional (3D) bioprinting of biomaterials and even the building of 3D cardiac tissues that can be directly transplanted to the damaged area. As an example, Ho et al. have already printed a polycaprolactone (PCL) scaffold using a microextrusion 3D printer and modified it with polycaprolactone CNT for cardiac tissue engineering. This composite scaffold made of PCL and polycaprolactone CNT better enhances cell proliferation and improves cardiomyoblast viability compared to non-modified PCL scaffold (128). Maiullari et al. have presented an innovative 3D bioprinting approach for vascularized cardiac tissue engineering. They have printed human umbilical vein endothelial cells (HUVECs) and iPSC-derived cardiomyocytes in various geometries. To encapsulate this multi-cellular construct, they have utilized an alginate/PEG-fibrinogen hydrogel scaffold owing to its enhanced capacity for damaged cardiac repair. As a result, they have demonstrated the feasibility of the construct for enabling the maturation of the cells into functional vascularized tissue in the heart (129). Basara et al. have shown that when Ti_3_C_2_T_x_ MXene nanosheets were bio-printed together with PEG, they can improve synchronous beating and conduction velocity during cardiomyocyte differentiation (102). Bioprinting applications have shown success in the regeneration of various other tissues. Yang et al. have implemented photothermal therapy through bio-printing bioglass-BP nanosheets composite scaffold for osteosarcoma and bone regeneration (130). Mehrotra et al. have designed a hybrid 3D construct using conductive non-mulberry silk-based bio-ink comprising CNTs to print vascularized CPs. Then, they also used microspheres loaded with IL-10, in order to immunomodulate the native MI environment with IL-10 delivery. They have concluded that IL-10 loaded microspheres increase oxygen availability and modulate the macrophages into anti-inflammatory M2 phenotype (131). It is a promising immunomodulatory study for the transplantation of biomaterial-based 3D construct into rat MI models for disease therapy. However, 3D printed tissues may still have poor perfusion problems, causing non-continuous viability of cells embedded on 3D printed tissue construct and thus tissue rejection (132, 133). Hence, it is foreseen that future studies will focus on 3D bioprinting of candidate nanomaterials and cells together to best match the cardiac tissue. Besides, the cross-talk studies of scientists, biomedical engineers, and cardiologists will reveal which of these approaches provide the most significant treatment to reduce the need for an organ transplant and establish cardiac regeneration methods specifically developed for individual patients and improve the lives of millions globally.

**Figure 2:**
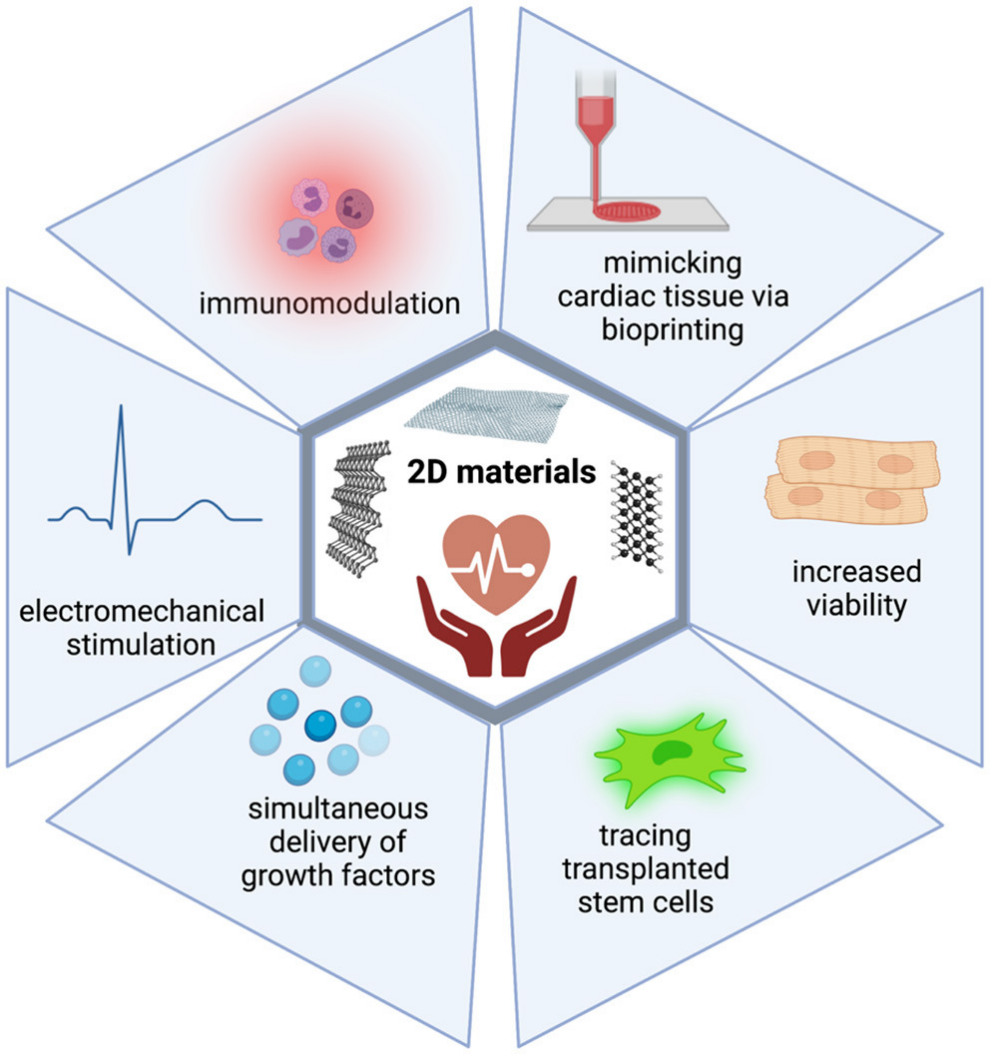
2D materials can overcome the limitations of current treatment strategies in CVDs. 2D materials harbor intrinsic properties which enable their use in cardiac regeneration. They can achieve better mimicking of cardiac tissue, improve viability of transplanted cells, allow better electromechanical integration, enhance immunomodulatory activities, trace transplanted cells, and deliver growth factors.

To conclude, thanks to their intrinsic properties, 2D materials can achieve better mimicking of cardiac tissue, improve the viability of transplanted cells, allow better electromechanical integration, enhance immunomodulatory activities, trace transplanted cells, and deliver growth factors (Figure 2). Therefore, it is clear that as new members of the 2D materials are explored, they will quickly become part of cardiac tissue engineering technologies.

## Author Contributions

AY coordinated the study. CGo and CGu performed literature search. All authors wrote the manuscript. All authors contributed to the article and approved the submitted version.

## Conflict of Interest

The authors declare that the research was conducted in the absence of any commercial or financial relationships that could be construed as a potential conflict of interest.

## Publisher's Note

All claims expressed in this article are solely those of the authors and do not necessarily represent those of their affiliated organizations, or those of the publisher, the editors and the reviewers. Any product that may be evaluated in this article, or claim that may be made by its manufacturer, is not guaranteed or endorsed by the publisher.
